# Repetitive Passive Movement Modulates Corticospinal Excitability: Effect of Movement and Rest Cycles and Subject Attention

**DOI:** 10.3389/fnbeh.2019.00038

**Published:** 2019-03-01

**Authors:** Shota Tsuiki, Ryoki Sasaki, Manh Van Pham, Shota Miyaguchi, Sho Kojima, Kei Saito, Yasuto Inukai, Naofumi Otsuru, Hideaki Onishi

**Affiliations:** ^1^Rehabilitation Center of Shiobara Hot Spring Hospital, Tochigi Medical Association, Tochigi, Japan; ^2^Institute for Human Movement and Medical Sciences, Niigata University of Health and Welfare, Niigata, Japan

**Keywords:** repetitive passive movement, duty cycle, conscious attention, motor evoked potential, corticospinal excitability

## Abstract

Repetitive passive movement (PM) affects corticospinal excitability; however, it is unknown whether a duty cycle which repeats movement and rest, or subjects’ conscious attention to movements, affects corticospinal excitability. We aimed to clarify the effect of the presence or absence of a duty cycle and subjects’ attention on corticospinal excitability. Three experiments were conducted. In Experiment 1, PM of the right index finger was performed for 10 min. Three conditions were used: (1) continuous PM (cPM) at a rate of 40°/s; (2) intermittent PM (iPM) with a duty cycle at 40°/s; and (3) iPM at 100°/s. In conditions 1 and 3, motor evoked potential (MEP) amplitude was significantly reduced. In Experiment 2, PM was performed for 30 min: condition 1 comprised cPM at a rate of 40°/s and Condition 2 comprised iPM at 40°/s. MEP amplitude significantly decreased in both conditions. In Experiment 3, PM was performed for 10 min: condition 1 comprised paying attention to the moving finger during iPM and Condition 2 was similar to Condition 1 but while counting images on a monitor without looking at the movement finger, and Condition 3 comprised counting images on a monitor without performing PM. MEP amplitude significantly increased only under Condition 1. Thus, afferent input from movements above a certain threshold may affect corticospinal excitability reduction. Furthermore, corticospinal excitability increases when paying attention to passive finger movement.

## Introduction

Corticospinal excitability decreases after passive movement (PM; Otsuka et al., 2017; Sasaki et al., 2017); this is thought to be due to post-exercise depression (PED). PED due to PM results in no changes to F waves, which are indicators of spinal cord excitability (Otsuka et al., 2017; Sasaki et al., 2017), but it increases short interval intracortical inhibition (SICI), which is an indicator of suppressive circuits in the cortex (Sasaki et al., 2017). Therefore, this phenomenon may be due to a decrease in the primary motor cortex (M1) activity. However, corticospinal excitability increases with PM (Macé et al., 2008) and remains unchanged (Lotze et al., 2003). This fluctuation in corticospinal excitability is thought to be influenced by differences in various stimuli such as the duration of movement, speed of movement, presence or absence of a duty cycle of repeated movement and rest, and presence or absence of a subject’s active attention on the movement.

In a previous study reporting that corticospinal excitability increased after PM (Macé et al., 2008), the authors used palm flexion and dorsiflexion of the wrist joint for 60 min with a duty cycle consisting of 5–8 s of rest after every 10 movements. In addition, their experiments were conditioned to focus on PM. In another previous study using peripheral electric stimulation (PES), intermittent stimulation with a duty cycle repeating stimulation and rest at an intensity above the motor threshold significantly increased corticospinal excitability (Chipchase et al., 2011). Moreover, corticospinal excitability significantly decreased with continuous theta burst stimulation (cTBS) but increased upon intermittent TBS (iTBS; Huang et al., 2005). From these previous studies, it appears that continuous and intermittent intervention with duty cycles of repeated stimulus and rest may have different effects on corticospinal excitability. Therefore, we hypothesized that corticospinal excitability would increase when setting a duty cycle in repetitive PM, and the purpose of Experiment 1 was to clarify the effect of the presence or absence of a duty cycle of repetitive PM on corticospinal excitability.

In the Experiment 2, the influence of number of movements was examined in order to clarify the effects of continuous repetitive PM and intermittent repetitive PM on corticospinal excitability. Therefore, the movement time was set to 30 min, and the number of the movements was increased.

In addition, attention and movement are closely related. For example, directing attention to the stimulating side during paired associative stimulation (PAS) intervention significantly increases corticospinal excitability (Stefan et al., 2004). However, corticospinal excitability does not change when focusing on the hand opposite to the stimulating side during PAS intervention (Stefan et al., 2004), and SICI decreases when paying attention to fingers during movement (Thomson et al., 2008). Furthermore, corticospinal excitability increases without F wave changes when attention is paid to the target hand during repetitive transcranial magnetic stimulation (rTMS) intervention (Conte et al., 2007, 2008). Additionally, attention to vibration stimulation increases corticospinal excitability and decreases SICI; however, corticospinal excitability does not change when attention is not paid (Rosenkranz and Rothwell, 2004).

These previous studies suggested that attention to the stimulated side during an intervention diminishes the activity of suppressive circuits in the cortex and thus increases corticospinal excitability. Therefore, in Experiment 3, we hypothesized that directing attention to the PM of index fingers would induce an increase in corticospinal excitability, and we aimed to clarify the influence of paying attention to repetitive PM on corticospinal excitability.

## Materials and Methods

### Subjects

A total of 19 healthy adults (16 males; age, 24.7 ± 6.0 years [mean ± standard deviation]; 17 right-handed) participated in this study. Experiment 1 utilized 15 healthy adults (13 males; age, 24.7 ± 6.6 years; 13 right-handed); Experiment 2 utilized 10 healthy adults (eight males; age, 24.0 ± 4.8 years; eight right-handed); and Experiment 3 utilized 14 healthy adults (12 males; age, 25.4 ± 6.6 years; 12 right-handed). No subjects had any central neurological or psychological disorders. This study was approved by the Ethics Committee of Niigata University of Health and Welfare and was conducted in accordance with the Declaration of Helsinki. All participants provided written informed consent before participating in this research.

### Electromyography (EMG)

The target muscle was the right first dorsal interosseous muscle (FDI), which was monitored with disposable Ag/AgCl electrodes in a belly−tendon montage. The earth electrode was wrapped around the right forearm. Electromyography (EMG) data were recorded using a surface electrode (Blue sensor, Metz) connected to an amplifier (×100; A-DL-720-140, 4 Assist, Tokyo, Japan). The amplified EMG signal was digitized using an A/D converter (Power Lab 8/30, AD Instruments, Colorado Springs, CO, USA). The sampling frequency was 4 kHz, and filtering was also performed using a 20 Hz high-pass filter. Data was imported into a personal computer and stored using analysis software (LabChart 7, AD Instruments).

### Motor Evoked Potential (MEP) Measurement

Motor evoked potential (MEP) was measured by TMS as a means of evaluating corticospinal excitability. A Magstim 200 (Magstim, Dyfed, UK) and a figure 8 coil (diameter, 9.5 cm) were used for MEP measurement. The coil was placed tangentially to the scalp, and the handle part of the coil was placed tangentially at approximately 45° behind the midline. The magnetic stimulation site was the finger area on the left M1 and was defined as the site (hot spot) where the MEP was most induced by the right FDI. Magnetic resonance imaging and Visor 2 TMS Neuronavigation (eemagine Medical Imaging Solutions GmbH, Berlin, Germany) were also used for identification of the hot spot on the right FDI. The position, direction and angle of the coil were made constant before and after the intervention. The magnetic stimulation intensity was defined as the intensity at which MEP amplitude was induced to about 1 mV at rest. MEP was measured 15 times before intervention (Pre) as well as immediately and 5 and 10 min after intervention (Post-0, Post-5, and Post-10, respectively). The magnetic stimulation interval was set to 5–6 s.

### Passive Movement

A custom-made PM control device (Takei Kiki kogyo, Niigata, Japan) that can control motion speed and angle was used in all three experiments.

### Experiment 1

**Figure 1:**
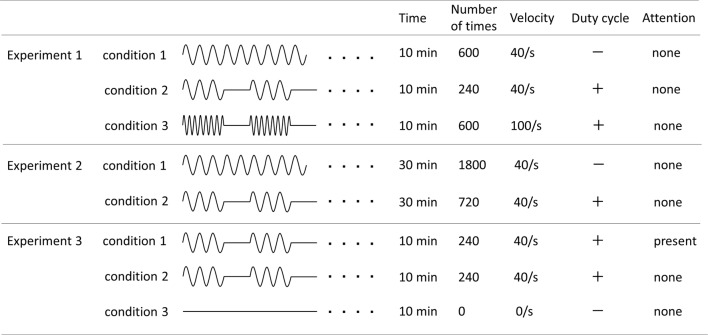
Details of all experimental conditions. Details regarding time of movement, number of movement, angular velocity, and presence or absence of duty cycle and attention of each experimental condition are indicated.

Three PM conditions were used (Figure 1). The movements examined were repetitive abduction–adduction movements of the right index finger from 0° to 20° abductions of the metacarpophalangeal (MP) joint for 10 min. Zero position was defined as the intermediate position of the MP joint. In Condition 1, angular velocity was 40°/s and the movement was continuously repeated (continuous PM−600; cPM_600). In Condition 2, intermittent PM−240 (iPM_240), consisted of PM for 4 s and rest for 6 s with an angular velocity of 40°/s. In Condition 3, intermittent fast PM−600 (ifPM_600), consisted of 4 s of movement followed by 6 s of rest was. However, to produce the same number of movements as in Condition 1, the angular velocity was set to 100°/s.

### Experiment 2

The PM condition were used and based on Condition 1 and Condition 2 in Experiment 1; however, both movement times were increased to 30 min (Figure 1). Therefore, in this case, Condition 1 utilized a cPM (cPM_1,800), whereas Condition 2 was an iPM with duty cycle of 4 s of movement followed by 6 s of rest (iPM_720).

### Experiment 3

Focusing on the presence or absence of the subject’s attention, three conditions were utilized (Figure 1). Condition 2 of Experiment 1 was slightly modified to produce Condition 1. In this case (attention), the subject was asked to observe and count the number of movements of the finger performing PM. Movement was set to 3, 4, or 5 s followed by 6 s of rest, and each of these cycles was randomly performed in 20 sets during the test. Instructions to the subject were as follows: “Please look at your right index finger and count how many times it moves. There will be a break of 6 s between each set of movements, at which point please tell me how many times your finger moved. Please repeat this for 10 min.”

In Condition 2 (no attention), PM was the same as in Condition 1 but subjects were asked to count the number of circles displayed on a monitor placed directly in front of their faces.

**Figure 2:**
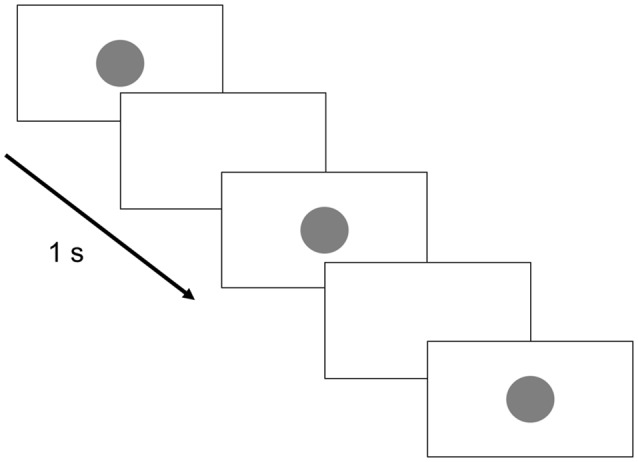
Monitor display. The circle presentation time was set to 0.5 s, whereas the circle presentation interval was set to 1 s. Three to five circles were randomly presented 20 times each. The break time between groups of circle displays was 6 s.

The image sequence displayed on the monitor is shown in Figure 2. Briefly, a perfect circle was presented for 0.5 s every 1 s and randomly blinked 3–5 times. This was followed by a white screen for 6 s and the process was then repeated. The subjects were given the following instructions: “Please count the number of blinking circles presented on the monitor. The screen will then turn white for 6 s, and during that time, please tell me how many times the circle flashed. Please repeat this for 10 min.”

In the Condition 3 (control), no PM was performed, and similar to Condition 2, the number of circles randomly displayed on the monitor was counted.

### Experimental Procedure (Figure 3)

In Experiments 1, 2, and 3, 15 MEPs were measured for Pre, Post-0, Post-5, and Post-10 using TMS. All the experiments were performed in the afternoon, and each condition was randomly assigned on a different day. In all experiments, the subjects sat in a reclining chair to which a headrest was attached, with their right forearms on a table while maintaining a comfortable posture at all times.

### Data Analysis

LabChart 7 software (AD Instruments) was used for MEP analysis. The maximum and minimum values of the 15 MEP waveforms obtained before and after the intervention for each condition were excluded, and the MEP amplitudes of the remaining 13 waveforms were averaged. The peak-to-peak value was then calculated as the MEP amplitude value.

### Statistical Analysis

PASW statistical analysis software Ver. 21 (SPSS; IBM, Armonk, NY, USA) was used for statistical analysis. Two-way repeated measure analysis of variance (ANOVA) was used to compare MEPs between INTERVENTION and TIME factors (Pre, Post-0, Post-5, Post-10) in each experiment. Mauchly’s test of sphericity was used to analyze the sphericity of the data obtained in each experiment. When the Mauchly’s test of sphericity could not be assumed, the Greenhouse–Geisser correction statistic was used. When a main effect or interaction was observed, multiple comparisons were performed using the Bonferroni method. The level of significance was set at 5%.

## Results

### Experiment 1

Changes in MEP over time are shown in Figure 4. Two-way repeated-measures ANOVA showed a significant main effect of the INTERVENTION factor (*F*_(2,28)_ = 5.019, *P* = 0.014, partial *η*^2^ = 0.264), of the main effect of the TIME factor (*F*_(2.036,28.500)_ = 5.443, *P* = 0.010, partial *η*^2^ = 0.280) and of their interaction (*F*_(6,84)_ = 3.277, *P* = 0.006, partial *η*^2^ = 0.190). Results of *post hoc* test showed that there was significant decrease in MEP in Post-0 and Post-5 compared with Pre in Condition 1 (*P* < 0.01) and 3 (*P* < 0.05), whereas there was no significant change in MEP in Condition 2.

**Figure 3:**
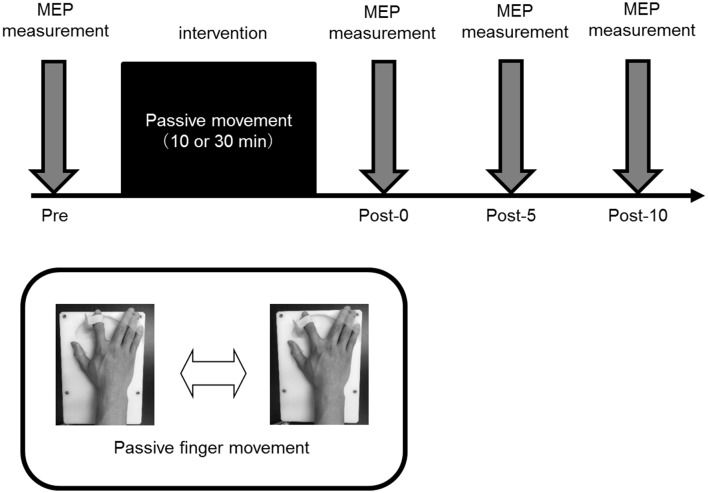
Experimental procedure. All the experiments were performed in the afternoon, and all participating subjects performed randomly assigned intervention tasks on different days. In all experiments, 15 motor evoked potentials (MEPs) were measured before intervention (Pre), and immediately (Post-0) and 5 (Post-5) and 10 min (Post-10) after the intervention.

**Figure 4:**
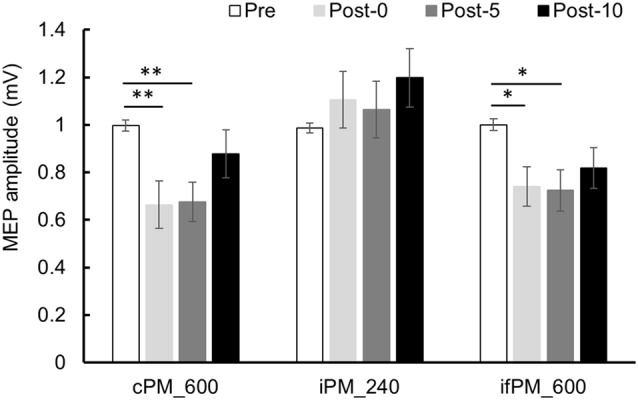
MEP amplitude before and after the intervention at Experiment 1. Mean MEP amplitude (mean ± standard error; SE) Pre, Post-0, Post-5, and Post-10. In the cPM_600 condition, MEP amplitude decreased significantly between Post-0 and Post-5 compared with Pre (*P* < 0.01). In the ifPM_600 condition, MEP amplitude decreased significantly between Post-0 and Post-5 compared with Pre (*P* < 0.05). In contrast, the iPM_240 condition did not result in any significant change in MEP amplitude before or after the intervention. *Post hoc* Bonferroni test. *P < 0.05, **P < 0.01.

### Experiment 2

**Figure 5:**
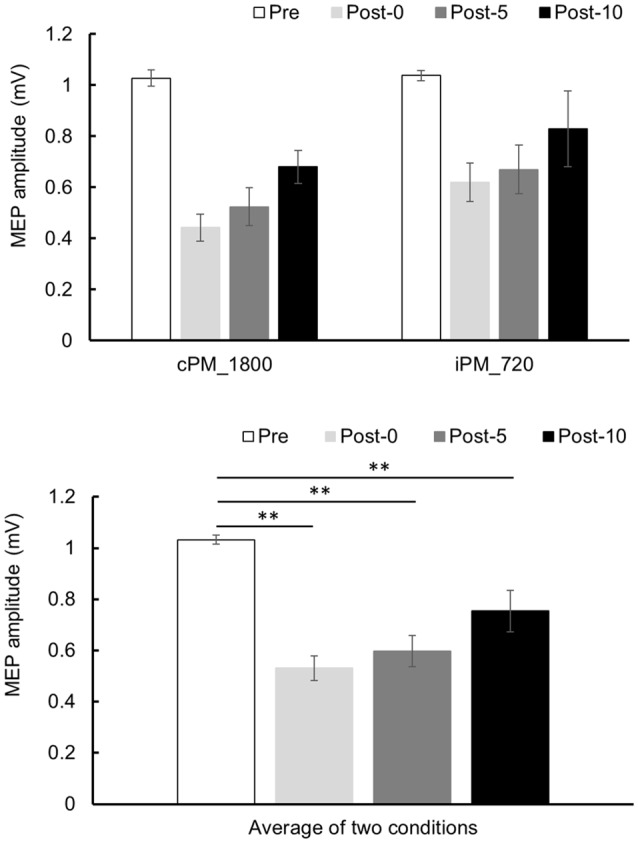
MEP amplitude before and after the intervention at Experiment 2. Mean MEP amplitude (mean ± SE) at Pre, Post-0, Post-5, and Post-10 (upper panel). Two-way repeated measure analysis of variance (ANOVA) showed that the main effect in the TIME factor was significant, and a *post hoc* test was conducted on the average results for the two conditions (lower panel). As a result, MEP amplitude decreased significantly between Post-0, Post-5, and Post-10 compared with Pre (*P* < 0.01). *Post hoc* Bonferroni test. **P < 0.01.

Changes in MEP over time are shown in Figure 5. Two-way repeated-measures ANOVA revealed a significant difference of the main effect of the TIME factor (*F*_(2.004,18.040)_ = 23.652, *P* < 0.001, partial *η*^2^ = 0.724), but no of the main effect of the INTERVENTION factor (*F*_(1,9)_ = 1.729, *P* = 0.221, partial *η*^2^ = 0.161) or their interaction (*F*_(3,27)_ = 0.632, *P* = 0.601, partial *η*^2^ = 0.066). Because the main effect of the TIME factor was significant, *post hoc* test was performed using the average results from the two conditions and found a significant decrease in MEP in Post-0, Post-5, and Post-10 compared with Pre (*P* < 0.01).

### Experiment 3

**Figure 6:**
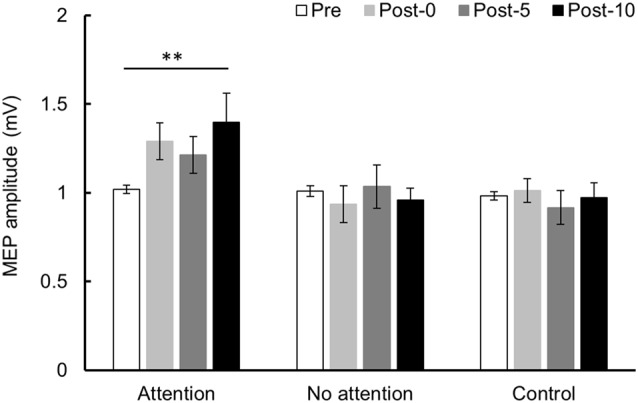
MEP amplitude before and after the intervention at Experiment 3. Mean MEP amplitude (mean ± SE) at Pre, Post-0, Post-5, and Post-10. When paying attention, the MEP amplitude increased significantly at Post-10 compared with Pre (*P* < 0.01). In contrast, when not paying attention and in the control condition, there was no significant change in MEP amplitude before and after the intervention. *Post hoc* Bonferroni test. **P < 0.01.

Changes in MEP over time are shown in Figure 6. Two-way repeated-measures ANOVA showed a significant difference in the main effect of the INTERVENTION factor (*F*_(2,26)_ = 5.079, *P* = 0.014, partial *η*^2^ = 0.281) and the interaction between INTERVENTION and TIME (*F*_(6,78)_ = 2.858, *P* = 0.014, partial *η*^2^ = 0.180) but no significant difference in main effect of the TIME factor (*F*_(3,39)_ = 1.678, *P* = 0.188, partial *η*^2^ = 0.114). Results of *post hoc* test showed that Condition 1 exhibited a significant increase in MEP amplitude at Post-10 compared with Pre (*P* < 0.01), but no significant MEP amplitude changes were observed in Conditions 2 and 3.

The number counted in all conditions was 240. In Conditions 1 and 3, only one person made a mistake in the count number once. In Condition 2, no subject made a mistake in the count number.

## Discussion

This study investigated the effect of the presence or absence of a duty cycle, which provides movement and rest during repetitive PM, and the influence of paying attention to PM on corticospinal excitability. As a result, in Experiment 1, corticospinal excitability decreased over 600 continuous repetitive PMs and 600 intermittent PMs. However, corticospinal excitability did not change over 240 intermittent repetitive PMs. The cortical excitability temporarily declines after light repetitive voluntary movement (Zanette et al., 1995; Teo et al., 2012a; Miyaguchi et al., 2013, 2016, 2017) and repetitive PM (Miyaguchi et al., 2013; Otsuka et al., 2017; Sasaki et al., 2017). During the PED period, spinal excitability does not change (Zanette et al., 1995; Otsuka et al., 2017; Sasaki et al., 2017) whereas SICI increases (Teo et al., 2012a; Sasaki et al., 2017). Therefore, it is considered that the reduction of corticospinal excitability after continuous or intermittent repetitive PMs in this experiment was due to decreased M1 excitability as previous reports (Otsuka et al., 2017; Sasaki et al., 2017). After intermittent iterative PM with the duty cycle, corticospinal excitability decreased with after a total of 600 movements but did not after 240 movements. Therefore, a minimum number of PMs may be required to induce corticospinal excitability decline. The intermittent repetitive passive and continuous repetitive movement conditions both contained 600 movements, and thus the proprioceptive afferent input was equivalent to under both conditions. In contrast, in the intermittent iterative PM, where movement occurred 240 times, the inherent proprioceptive afferent input was possibly insufficient to induce changes in corticospinal excitability. Previous studies have reported that M1 acts not only in voluntary movements but also in PMs (Weiller et al., 1996; Terumitsu et al., 2009; Onishi et al., 2013). The activity of M1 is thought to be induced by the proprioceptive somatosensory input accompanying PM (Reddy et al., 2001). Moreover, proprioceptive inputs from muscles and joints reach not only the primary somatosensory cortex (S1) but also M1 (Lucier et al., 1975; Zarzecki et al., 1978; Onishi et al., 2011). Therefore, repetitive activity of M1 due to repetitive PM may have changed corticospinal excitability. However, in order to induce corticospinal excitability changes after PM, a minimum number of repetitive activities of M1 may be necessary. Therefore, we hypothesized that proprioceptive somatosensory input due to a number of movement cycles above a certain threshold affects corticospinal excitability, and Experiment 2 was performed to test this.

Experiment 2 used two conditions from Experiment 1, those being continuous repetitive PM, where corticospinal excitability decreased, and intermittent repetitive PM, where corticospinal excitability did not change, and movement time was increased from 10 to 30 min. Therefore, continuous repetitive PM consisted of 1,800 movements overall, whereas intermittent repetitive PM consisted of 720 movements. As a result, corticospinal excitability decreased in both conditions, indicating that corticospinal excitability decreases with increasing numbers of movements, even with intermittent repetitive PMs where corticospinal excitability did not change with 240 movement repeats. Therefore, it seems that changes in corticospinal excitability do not depend on the presence or absence of the duty cycle but rather are influenced by the number of movements. It is reported that the cortical excitability temporarily decreases after repetitive PM (Miyaguchi et al., 2013; Otsuka et al., 2017; Sasaki et al., 2017). This phenomenon is considered as PED and potential mechanisms of PED include long-term depression, reduced neurotransmitter levels, decreased excitability of intracortical glutamatergic networks, or increased excitability of inhibitory GABAergic networks (Zanette et al., 1995; Samii et al., 1996; Teo et al., 2012b). It is suggested that the decline in MEP in Experiments 1 and 2 is due to PED. However, the influence of movement number of PM engaging in the neurophysiological mechanism remains unknown and this is the limitation of our study. From now on, it is necessary to clarify detailed evaluation and mechanism in movement number of PM.

The results from the first two experiments suggested that the presence or absence of a duty cycle does not affect changes in corticospinal excitability. Therefore, in Examination 3, we examined the influence of paying attention to PMs on corticospinal excitability. As a result, when attention was paid to the moving finger during PM, corticospinal excitability increased, whereas corticospinal excitability did not change under conditions where attention was not directed to passive finger movements. Macé et al. (2008) reported that corticospinal excitability increases after 60 min of repetitive PM of the wrist joint. In their study, subjects’ attention was directed to the PM. In other earlier studies on the effect of attention on corticospinal excitability, corticospinal excitability is significantly increased by directing attention to the target hand during PAS intervention (Stefan et al., 2004), during finger movements (Thomson et al., 2008), during rTMS intervention (Conte et al., 2007, 2008), and during vibration stimulation (Rosenkranz and Rothwell, 2004). There is evidence that neural activity can be affected by changes in attention at a low-level cortical output stage in the M1 (Baker et al., 1999; Rösler et al., 1999). It has been shown that attention related physiological events may influence cortical plasticity changes in S1 (Buchner et al., 1999), and results of the study by Stefan et al. (2004) extend these observations to the motor cortex (Stefan et al., 2004). It is also reported that short-latency afferent inhibition (SAI) is decreased by attention (Mirdamadi et al., 2017). SAI (Tokimura et al., 2000) provides a method to investigate the modulatory effects of somatosensory afferent on motor cortex excitability. In addition to GABA_A,_ central cholinergic are involved in generation of SAI (Ziemann et al., 2015). It is further reported that SICI is decreased by attention. It is suggested that SICI is involved in the inhibition mechanism of GABA system (Ziemann et al., 1996a,b). Thus, the attention task in this study might also have contributed to the reduction in GABA system and choline system suppression circuit in M1. Therefore, it is possible that corticospinal excitability increased in this study as well as in the previous study when attention was paid to passive finger movements. However, since detailed mechanism is unknown in this research, further investigation is necessary.

This study demonstrated that continuous repetitive PMs and intermittent repetitive PMs consisting of a total number of 600 movements reduced corticospinal excitability but a total of 240 intermittent repetitive PMs did not. Furthermore, 1,800 continuous repetitive PMs and 720 intermittent repetitive passive exercises decreased corticospinal excitability. Therefore, corticospinal excitability was affected by afferent input based on the number of movements above a certain threshold. Furthermore, corticospinal excitability increased when subjects directed their attention to the moving finger during PM.

## Data Availability

Anonymized data for the manuscript are available on request. Please contact the corresponding authors for the same.

## Author Contributions

HO and ST conceived the study and designed the experiments. ST, RS and MP performed the experiments and performed statistical analysis. SM and SK performed data interpretation. KS, YI and NO helped in writing the manuscript. HO and ST wrote the manuscript. All authors have read and approved the final manuscript.

## Conflict of Interest Statement

The authors declare that the research was conducted in the absence of any commercial or financial relationships that could be construed as a potential conflict of interest.

## Abbreviations

MEP: Motor evoked potential
TMS: Transcranial magnetic stimulation
PED: Post-exercise depression
SICI: Short interval intracortical inhibition
SAI: Short-latency afferent inhibition
M1: Primary motor cortex
PES: Peripheral electric stimulation
TBS: Theta burst stimulation
cTBS: continuance TBS
iTBS: intermittent TBS
PAS: Paired associative stimulation
rTMS: Repetitive transcranial magnetic stimulation
EMG: Electromyography
FDI: First dorsal interosseous
PM: Passive movement
S1: Primary somatosensory cortex
ANOVA: Analysis of variance
cPM: continuous passive movement
iPM: intermittent passive movement
ifPM: intermittent fast passive movement.
